# Automated Cone Beam Computed Tomography Segmentation of Multiple Impacted Teeth With or Without Association to Rare Diseases: Evaluation of Four Deep Learning‐Based Methods

**DOI:** 10.1111/ocr.12890

**Published:** 2025-01-02

**Authors:** Eloi Sinard, Laurent Gajny, Muriel de La Dure‐Molla, Rufino Felizardo, Gauthier Dot

**Affiliations:** ^1^ UFR Odontologie Université Paris Cité Paris France; ^2^ Service de Medecine Bucco‐Dentaire AP‐HP, Hopital Pitie Salpetriere Paris France; ^3^ Institut de Biomecanique Humaine Georges Charpak Arts et Metiers Institute of Technology Paris France; ^4^ Reference Center for Oral and Dental Rare Disease, ORARES AP‐HP, Rothschild Hospital Paris France

**Keywords:** computer‐assisted, cone beam computed tomography, deep learning, impacted, orthodontics, surgery, tooth

## Abstract

**Objective:**

To assess the accuracy of three commercially available and one open‐source deep learning (DL) solutions for automatic tooth segmentation in cone beam computed tomography (CBCT) images of patients with multiple dental impactions.

**Materials and Methods:**

Twenty patients (20 CBCT scans) were selected from a retrospective cohort of individuals with multiple dental impactions. For each CBCT scan, one reference segmentation and four DL segmentations of the maxillary and mandibular teeth were obtained. Reference segmentations were generated by experts using a semi‐automatic process. DL segmentations were automatically generated according to the manufacturer's instructions. Quantitative and qualitative evaluations of each DL segmentation were performed by comparing it with expert‐generated segmentation. The quantitative metrics used were Dice similarity coefficient (DSC) and the normalized surface distance (NSD).

**Results:**

The patients had an average of 12 retained teeth, with 12 of them diagnosed with a rare disease. DSC values ranged from 88.5% ± 3.2% to 95.6% ± 1.2%, and NSD values ranged from 95.3% ± 2.7% to 97.4% ± 6.5%. The number of completely unsegmented teeth ranged from 1 (0.1%) to 41 (6.0%). Two solutions (Diagnocat and DentalSegmentator) outperformed the others across all tested parameters.

**Conclusion:**

All the tested methods showed a mean NSD of approximately 95%, proving their overall efficiency for tooth segmentation. The accuracy of the methods varied among the four tested solutions owing to the presence of impacted teeth in our CBCT scans. DL solutions are evolving rapidly, and their future performance cannot be predicted based on our results.

**Abbreviations**
3D: three‐dimensional
CBCT: cone‐beam CT
DL: deep learning
DSC: Dice similarity coefficient
GPU: graphics processing unit
MPR: multi‐planar reconstructions
NSD: normalized surface distance

## 1 Introduction

Multiple dental impactions are complex clinical situations that often require a multidisciplinary approach involving both orthodontists and oral surgeons. The clinical decision‐making process requires precise three‐dimensional (3D) visualization to study the relationship between impacted teeth and adjacent anatomical structures (such as roots and sinuses). Cone beam computed tomography (CBCT) is recommended for refining diagnoses and establishing treatment plans [1, 2, 3]. Impacted teeth may be linked to rare diseases that affect the oral cavity, such as cleidocranial dysplasia, porencephaly, osteopetrosis and Pierre Robin syndrome.

To date, CBCT scans have mainly been analysed in the clinic using multi‐planar reconstruction (MPR) and 3D threshold‐based renderings. Unfortunately, these 3D renderings are often difficult to analyse because of the low‐contrast resolution of CBCT scans and the frequent presence of artifacts [4]. CBCT segmentation is a key step in most digital dentistry workflows, aimed at individualizing bone and tooth structures and creating patient‐specific 3D digital model [5, 6]. Particularly in the case of multiple impacted teeth, these patient‐specific models could facilitate orthodontic diagnosis, movement planning, multidisciplinary discussion, research and communication with the patient [7].

Manual tooth segmentation process has been reported to require between 1.5 and 3.5 h [8, 9]. Recently, deep learning (DL)‐based methods have been proposed to perform this segmentation fully automatically within a few minutes, with results reported to be equivalent to or even superior to those of an experienced operator [10, 11, 12, 13, 14]. Several commercial and open‐source solutions are available for automatic DL tooth segmentation from CBCT scans. However, these DL methods have never been specifically evaluated on CBCT scans of patients with multiple dental impactions, and some research reports on DL methods have explicitly excluded patients with impacted teeth from their datasets. This is a critical issue, as DL methods may demonstrate excellent performance when evaluated on internal datasets but yield poorer results when tested under real clinical conditions [15].

The aim of this study was to evaluate the accuracy of three commercially available and one open‐source DL solution for automatic tooth segmentation on CBCT images of patients with multiple dental impactions.

## 2 Methods

### 2.1 Patient Selection

Data were randomly selected from a retrospective cohort of patients treated at a public hospital's dental department between 2015 and 2023. Patients of all age groups were included. The inclusion criteria required participants to have a maxillary and/or mandibular CBCT scan displaying multiple dental impactions (*n* ≥ 2), regardless of whether they were related to a rare disease. An impacted tooth was defined as one that had not erupted on an arch within the expected developmental window. The exclusion criterion was the refusal to participate in the study (patients were contacted via mail). Finally, 20 patients (20 CBCT scans) were included in this study. All CBCT scans were part of the patients' treatment, and no scans were performed specifically for this study. This study was approved by the Sorbonne Université Institutional Review Board, and all patients consented to participate in this study.

The mean voxel size of the CBCT scans was 0.2 mm^3^. Most scans (*n* = 16, 80%) were acquired using a PLANMECA Promax mid‐CBCT device (Helsinki, Finland). Fourteen (70%) CBCT scans involved the maxillary–mandibular region; five (25%), the maxillary region and one (5%), the mandibular region. Characteristics of the CBCT scans are listed in Table 1.

**TABLE 1:** Characteristics of the CBCT scans in the dataset.

Number (%)	Machine	Voxel size (mm^3^)	Field of view
12 (60)	PLANMECA Promax mid	0.15–0.3	Maxillary–mandibular region
3 (15)	PLANMECA Promax mid	0.15–0.3	Maxillary region
1 (5)	PLANMECA Promax mid	0.15–0.3	Mandibular region
1 (5)	Instrumentarium orthopantomograph OP300	0.35	Maxillary–mandibular region
1 (5)	Newtom hybplus	0.15	Maxillary region
1 (5)	General electrics medical systems revolution EVO	0.3	Maxillary region
1 (5)	NewTom NTVGiEVO	0.2	Maxillary–mandibular region

### 2.2 Patient Characteristics

Age, sex, presence of any associated rare disease and the number of impacted and supernumerary teeth were recorded for each patient.

### 2.3 Expert Segmentation Process (Reference Test)

Reference segmentation was performed by experts employing the following semi‐automated process proposed in previously published studies [16, 17, 18]. Each CBCT scan was processed using a pretrained, previously published nnU‐Net model [13, 19]. The segmentation masks of the maxillary and mandibular teeth were then exported to 3D Slicer software (v5.6) [20] for manual correction. The correction was conducted by a first expert (a trained orthodontic resident) [step 1], followed by slice‐by‐slice verification by a second expert (an orthodontist with more than 5 years of experience in 3D image evaluation) [step 2]. Steps 1 and 2 were repeated until step 2 was validated. The time required for manual refinement ranged from 20 to 240 min for each CBCT scan. This process resulted in two segmentation masks: (1) maxillary teeth and (2) mandibular teeth.

### 2.4 DL‐Based Segmentation (Index Test)

For each CBCT scan, four DL segmentations of the maxillary and/or mandibular teeth were automatically obtained.

1. The data were uploaded to the web‐based platform Diagnocat (DGNCT LLC, Miami, Florida USA; https://eu.diagnocat.com/) and segmented according to the manufacturer's recommendations between June 15 and 19, 2023. The segmentations of the maxillary and mandibular teeth were exported as individual STL files, corresponding to the number of teeth. These files were then merged by arch in the 3D Slicer software to create segmentation masks for the maxillary teeth and/or mandibular teeth.
2. The data were uploaded to the Relu (Relu, Leuven, Belgium) web‐based platform (https://creator.relu.eu/) and segmented according to the manufacturer's recommendations on September 1, 2023. The segmentations of the maxillary and mandibular teeth were exported as two STL files. These two files were then processed in 3D Slicer software to create segmentation masks for the maxillary and/or mandibular teeth. One file had a 3D coordinate error and was not included in the tests.
3. The data were uploaded to the CephX (Orca Dental Al, Las Vegas, Nevada USA) web‐based platform (https://cephx.com/) and segmented according to the manufacturer's recommendations between December 1 and December 22, 2023. The segmentations of the maxillary and mandibular teeth were exported as individual STL files, corresponding to the number of teeth. These files were then merged by arch in 3D Slicer software to create segmentation masks for the maxillary and mandibular teeth. Two files could not be imported into the platform and were excluded from the tests.
4. The data were segmented using the open‐source DentalSegmentator model [18] on March 20, 2024. The segmentation masks of the maxillary and mandibular teeth were exported.

### 2.5 Quantitative Assessment of DL Segmentations

To quantitatively assess the correlation between expert and DL segmentation, we compared the results of each tested method with those of expert segmentation for the maxillary and mandibular teeth segmentation masks.

We followed the best practice recommendations and used both volume‐ and surface‐based metrics. The closer the score was to 100%, the more closely the tested segmentation matched the reference segmentation. Our volume‐based metric was the Dice similarity coefficient (DSC), which is commonly used in the literature. Our surface‐based metric was the normalized surface distance (NSD) at 1 mm. The NSD has been shown to correlate significantly with the time required to correct the segmentation for clinical use [21]. For each method, we calculated the number of DL segmentations with an NSD score ≥ 95%, which has been proposed as a threshold for a segmentation to be considered clinically useful [21].

Additionally, to assess the proportion of missing teeth in the segmentations, we counted the number of completely unsegmented teeth for each method.

### 2.6 Qualitative Assessment of DL Segmentations

Three cases representative of our dataset were qualitatively evaluated through visual comparison with experts and DL segmentation.

### 2.7 Statistical Analysis

We statistically compared the four DL methods for the DSC and NSD metrics. We first assessed the normality of the residuals (based on the Shapiro–Wilk test) and performed an adapted ANOVA test, followed by multiple pairwise comparisons to determine which groups were different from each other.

To test the correlation between the number of supernumerary teeth and our NSD results, we first assessed the normality of the data using the Shapiro–Wilk normality test and then applied the Pearson correlation coefficient and Spearman's rank correlation coefficient tests for normal and non‐normal data distributions, respectively.

Values of *p* < 0.05 were considered statistically significant. All data were analysed using Python (v3.7) and R Statistical Software (v4.2.2; R Core Team 2022) [22].

## 3 Results

### 3.1 Patient Characteristics

In our dataset, the mean age of the patients was 16 ± 6.5 years (minimum age 9 years, maximum age 31 years). Twelve patients (60%) had a rare disease with oral manifestations, including nine cases of cleidocranial dysplasia, one of porencephaly, one of osteopetrosis and one of Pierre Robin sequence. The mean number of impacted teeth was 12 ± 9, and the mean number of supernumerary teeth was 3 ± 6 (minimum 2, maximum 21; Table 2).

**TABLE 2:** Patient characteristics.

Characteristic	Value
Age, mean (±SD)	16.1 (±6.5)
Gender, no. (%)	
Female	13 (65)
Male	7 (35)
Presence of a rare disease with oral repercussions	
Yes	12 (60)
Cleidocranial dysplasia	9 (45)
Osteopetrosis	1 (5)
Porencephaly	1 (5)
Pierre Robin syndrome	1 (5)
No	8 (40)
Number of impacted teeth, mean (±SD)	11.7 (±9.8)
Supernumerary teeth, mean (±SD)	3.2 (±5.6)

### 3.2 Quantitative Assessment of DL Segmentations

**FIGURE 1:**
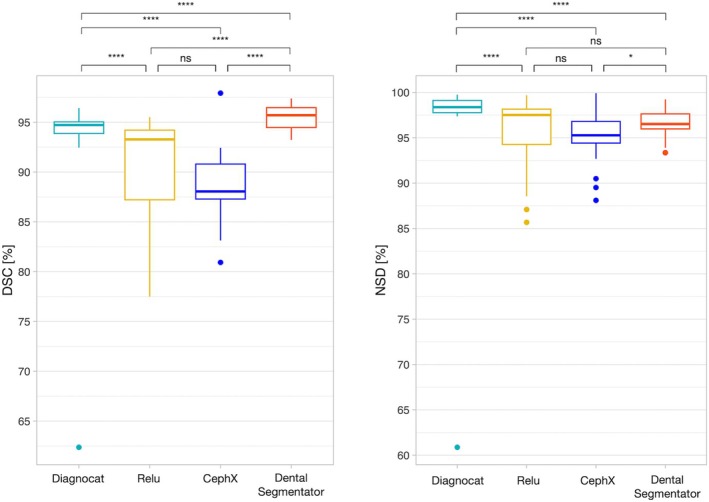
Distribution of DSC (left) and NSD (right) results and analysis of the statistical comparisons between the groups. ns: *p* > 0.05; *p ≤ 0.05; **p ≤ 0.01; ***p ≤ 0.001; **** *p* ≤ 0.0001.

Overall, DSC scores ranged from 88.5% ± 3.2% to 95.6% ± 1.2%, and NSD scores ranged from 95.3% ± 2.7% to 97.4% ± 6.5%. The results for each method are presented in Table 3, and Figure 1 shows their distribution. The number of DL segmentations with an NSD score ≥ 95% varied between the solutions: 97.1% (*n =* 33) for Diagnocat, 66.7% (*n =* 22) for Relu, 53.3% (*n =* 16) for CephX and 91.2% (*n =* 31) for DentalSegmentator. The number of completely unsegmented teeth differed according to the method tested: 0.9% (*n =* 8) for Diagnocat, 6.0% (*n =* 41) for Relu, 4.0% (*n =* 30) for CephX and 0.1% (*n =* 1) for DentalSegmentator.

**TABLE 3:** Average DSC and NSD results.

Measurement	Diagnocat	Relu	CephX	DentalSegmentator
DSC				
Mean (%)	93.6 95% CI [91.7, 95.6]	90.2 95% CI [88.4, 92.1]	88.5 95% CI [87.3, 89.7]	95.6 95% CI [95.2, 96]
SD (%)	5.6 95% CI [4.5, 7.4]	5.2 95% CI [4.1, 6.8]	3.2 95% CI [2.6, 4.3]	1.2 95% CI [1, 1.6]
NSD				
Mean (%)	97.4 95% CI [95.1, 99.6]	95.5 95% CI [94.1, 96.9]	95.3 95% CI [94.3, 96.4]	96.6 95% CI [96.1, 97.1]
SD (%)	6.5 95% CI [5.2, 8.5]	4.0 95% CI [3.3, 5.4]	2.7 95% CI [2.2, 3.7]	1.4 95% CI [1.1, 1.8]

Abbreviations: 95% CI, confidence interval at 95%; SD, standard deviation.

As the residuals were not normally distributed and there was missing data, we used the Skillings–Mack test for our ANOVA analysis, which is an adapted version of the Friedman test designed for datasets with missing values (*p* = 0.000001). We then performed multiple pairwise comparisons using the Wilcoxon signed‐rank test for paired data to determine which groups differed from each other. Based on the DSC metric, the DentalSegmentator method showed significantly superior results compared with the other three methods. Based on the NSD metric, the Diagnocat method showed significantly superior results compared with the other three methods. The results of the statistical tests are shown in Figure 1. When evaluating the correlation between the number of supernumerary teeth and the NSD results, only Relu method exhibited a statistically significant relationship (Spearman's correlation *r* (17) = 0.642, *p = 0*.003).

### 3.3 Qualitative Assessment of DL Segmentations

**FIGURE 2:**
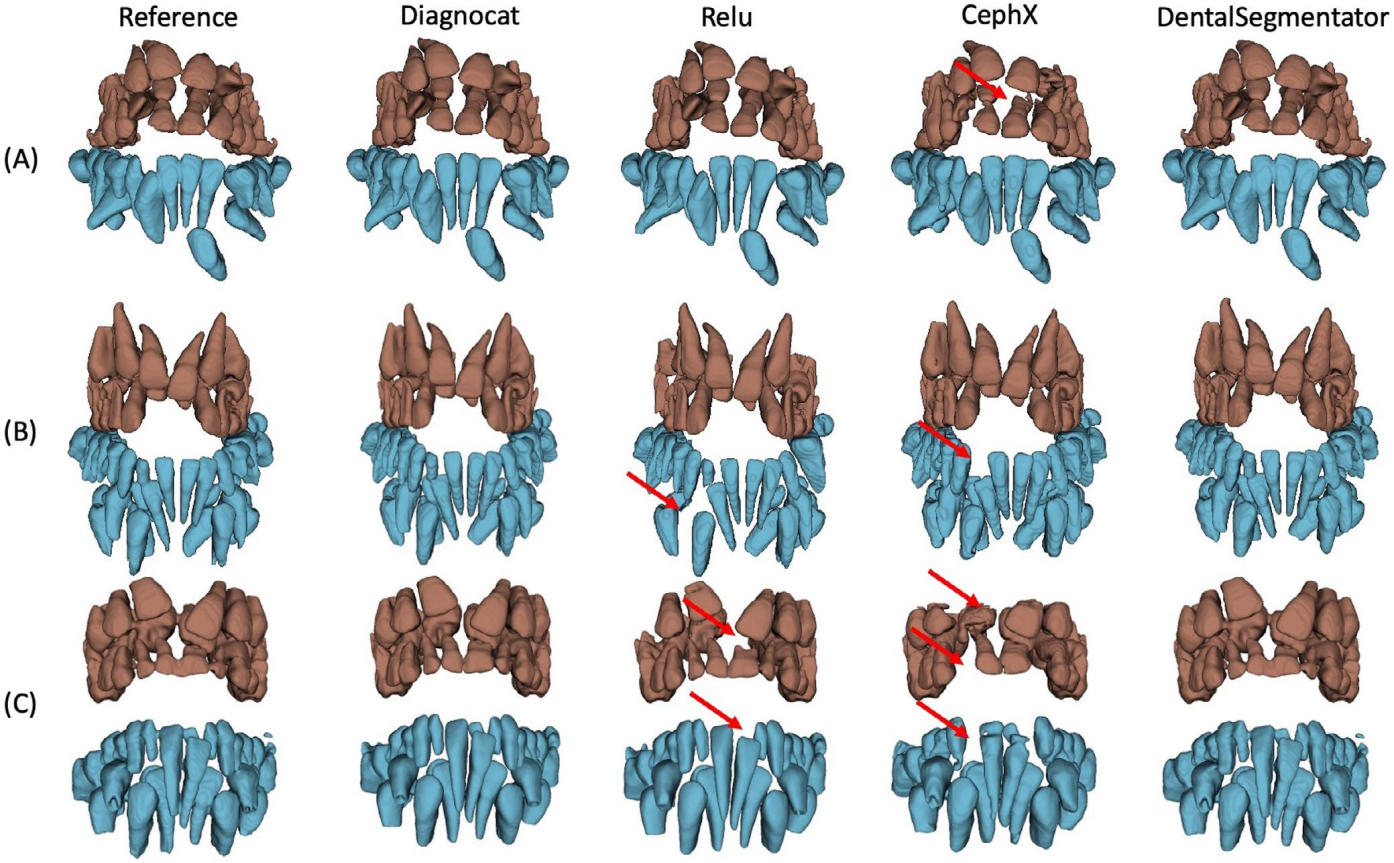
Three‐dimensional models of three representative cases (A, B and C) were reconstructed from reference and DL segmentations. The maxillary teeth are colored in red, while the mandibular teeth are in blue. Red arrows indicate under‐segmented areas.

**FIGURE 3:**
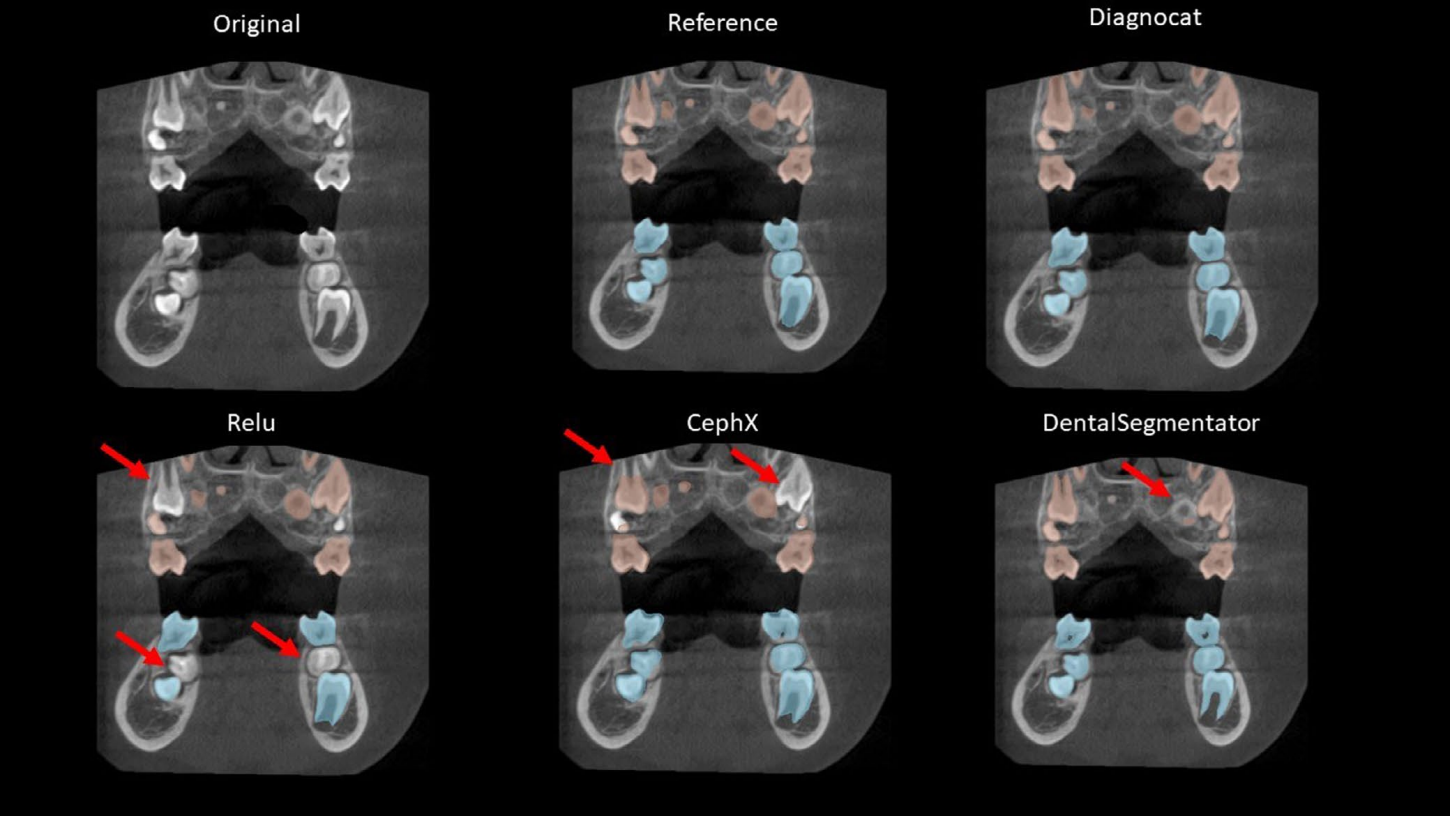
A representative case (Case C from Figure 2) showing a frontal section of the original CBCT, the reference segmentation and the DL segmentations. The maxillary teeth are colored in red, and the mandibular teeth are in blue. Red arrows signify under‐segmented regions.

Three cases representative of our dataset were selected to illustrate the results. The 3D models are shown in Figure 2, and Figure 3 depicts the CBCT frontal section of Case C. This qualitative assessment illustrates the under‐segmentations observed in some of the test DL segmentations, which can range from minor portions of a tooth to an entirely missing tooth (indicated by red arrows in the figures).

## 4 Discussion

The main objective of this study was to evaluate the accuracy of four DL‐based tooth segmentation solutions for CBCT scans of patients with multiple dental impactions. Our original dataset of 20 CBCT scans represented particularly challenging clinical situations, with an average of 12 impacted teeth per patient and some participants having as many as 21 impacted teeth. Notably, 12 (60%) of the individuals had a rare disease with orofacial implications; CBCT scans from such patients are often excluded from studies evaluating DL models. Our results indicated that all tested methods showed mean NSD results of approximately 95%, proving their overall efficiency for tooth segmentation. However, there were significant differences between the four tested solutions owing to the inclusion of CBCT scans from patients with impacted teeth. Diagnocat and DentalSegmentator showed better results than those of Relu and CephX in all tested parameters: DSC, NSD, number of DL segmentations with NSD ≥ 95% and number of completely unsegmented teeth. Using an NSD threshold of ≥ 95% to accept results for a patient, Diagnocat and DentalSegmentator had one and three failures, respectively, while Relu and CephX had 11 and 14 failures, respectively.

A direct comparison of our results with those in the literature is difficult, as this is the first study to focus on patients with multiple dental impactions. To our knowledge, only one previous study evaluated a DL model (Relu) for impacted canine segmentation on CBCT images [23]. The results of Diagnocat and DentalSegmentator are in line with those previously reported in the literature (for Diagnocat, intersection over union IoU = 0.96; for DentalSegmentator, DSC = 94% and NSD = 98%), demonstrating good generalizability of these solutions [11, 18, 24]. The results for Relu tend to be below those previously published in the literature (DSC = 99%) [14, 23, 25]. Several studies have evaluated the accuracy of the CephX method using linear measurements; however, we did not find any research reports on volumetric measurements [5, 26].

There are several possible explanations for these findings. First, the datasets used to train the algorithms of Diagnocat and DentalSegmentator may have included a greater number of CBCT scans with impacted and supernumerary teeth compared to those used for Relu and CephX. Our dataset included atypical clinical situations involving numerous impacted teeth and patients with rare diseases. The presence of unusual dental shape variations in these CBCT scans may have negatively affected the results of the algorithms that were not trained on these types of data. In addition, at the time of the study, the Relu algorithm included a classifier that did not account for supernumerary teeth, which may explain the statistical correlation observed between our results and the number of supernumerary teeth. Overall, our results highlight the importance of externally evaluating DL segmentation solutions in relation to the specific clinical situations in which they will be applied [15].

Cloud‐based commercial solutions have the advantages of being easily accessible and intuitive, particularly from a clinician's perspective. The clinician does not need to install any special software but simply upload the CBCT scan in DICOM format to the manufacturer's user‐friendly website, where the process is completed automatically within a few minutes. When we tested these solutions, the three online platforms did not provide the same user experience. Relu and CephX offered the option to overlay the segmentation on the CBCT slices for qualitative control, whereas Diagnocat did not. This absence could mislead users if certain regions were not correctly segmented. Relu also provided certain tools for manual correction of the segmentation masks. The procedure for correcting the CephX and Diagnocat segmentations added a time‐consuming post‐processing step that required different software and was unsuitable for novice users.

The open‐source DentalSegmentator solution is publicly available as a free 3D slicer extension that runs locally on a user's computer. Software installation is mandatory, and a compatible graphics processing unit is required for fast results. The segmentation results can be overlaid on the CBCT slices, and corrections can be made using the software. This solution is aimed primarily at craniofacial researchers, as the 3D Slicer software is not intended for clinical use and is less user‐friendly than commercial alternatives.

Depending on the intended clinical or research application of the segmentations, the required accuracy will vary, necessitating checks and corrections for any over‐ or under‐segmentation. For example, segmentation used to visualize teeth for treatment planning purposes does not require sub‐millimeter accuracy, as MPR slices are used to detect pathologies such as root resorption. However, 3D printing of an autotransplant guide requires verification and possible correction of the segmentation masks at the donor and recipient sites to obtain the most accurate guide possible [27].

Our study had several limitations. Our dataset comprised a small number of patients (*n =* 20), which may have limited the power of our study and the generalizability of the findings. Missing data, due to two upload failures for CephX and one 3D coordinate mismatch for Relu, might have affected our results and conclusions. Building a larger dataset is essential for further evaluation, as no public datasets currently exist. A greater variety of CBCT devices would be necessary to evaluate the generalizability of the solutions more closely. In addition, the three commercial solutions allowed for individual segmentation and classification of teeth, a feature not provided by the DentalSegmentator algorithm and, hence, not evaluated in this study. Finally, as these software programs are regularly updated, our results are only valid for the period during which the evaluations were performed.

## 5 Conclusion

All the tested methods showed a mean NSD of approximately 95%, proving their overall efficiency for tooth segmentation. The accuracy of the methods varied among the four tested solutions owing to the inclusion of CBCT scans from patients with impacted teeth. Diagnocat and DentalSegmentator showed better results than those of Relu and CephX across all tested parameters. These DL solutions are rapidly evolving, and their future performances cannot be predicted based on our results.

## Author Contributions

E.S. contributed to the conception, data acquisition, analysis and interpretation and drafted the manuscript. L.G. and G.D. contributed to the conception, data interpretation, performed statistical analysis and critically revised the manuscript. M.L.D.‐M. and R.F. contributed to the conception, data analysis and critically revised the manuscript. All authors approved the submitted version.

## Ethics Statement

This study was ethically approved by an Institutional Review Board (CER Sorbonne Université), and all patients consented to participate.

## Conflicts of Interest

The authors declare no conflicts of interest.

## Data Availability

The data that support the findings of this study are available from the corresponding author upon reasonable request.
